# Gender Equality Training for Students in Higher Education: Scoping Review

**DOI:** 10.2196/60061

**Published:** 2025-07-11

**Authors:** Claire Condron, Mide Power, Midhun Mathew, Siobhan Lucey, Patrick Henn, Tanya Dean, Michelle Kirrane Scott, Walter Eppich, Siobhan M Lucey

**Affiliations:** 1 RCSI SIM Royal College of Surgeons in Ireland University of Medicine and Health Sciences Dublin Ireland; 2 Department of Sociology School of Social Sciences and Philosophy Trinity College Dublin Dublin Ireland; 3 School of Medicine University College Cork Cork Ireland; 4 Conservatoire Technology University of Dublin Dublin Ireland; 5 Faculty of Medicine, Dentistry and Health Sciences Department of Medical Education & Collaborative Practice Centre The University of Melbourne Melbourne Australia

**Keywords:** gender equality, tertiary education, students, training, higher education institutions

## Abstract

**Background:**

Despite recent improvements, gender inequality persists within the higher education sector, as evidenced by the proportionally greater number of student and academic leadership positions occupied by male students and staff. Gender equality education and training for students may help to develop awareness, knowledge, and skills among individual students, building capacity to address biases and accelerate culture change in higher education institutions.

**Objective:**

We aimed to identify and explore the existing literature on gender equality training interventions for students in tertiary education, with a particular emphasis on training content, methodology, and outcome evaluation.

**Methods:**

The 6-stage framework developed by Arskey and O’Malley was used to map the literature related to current best practice in gender equality training for students in higher education. Systematic database searches of peer-reviewed literature were carried out and 3142 titles, 333 abstracts, and 52 full-text articles were screened for eligibility with 14 (27%) articles selected for inclusion in this review.

**Results:**

The selected studies detailed a range of pedagogical approaches, including didactic lectures, participatory and co-design workshops, reflective writing, and service-learning, with durations ranging from a single interaction to 1 year. Most articles reviewed did not explicitly state their study aims or research question, and the theoretical underpinnings were generally vaguely described. The longer-term impact of most interventions was unclear, as evaluation metrics seldom go beyond the level of adoption.

**Conclusions:**

This scoping review shows that the literature base for gender equality training for tertiary students lacks coherence, highlighting the need for further work to evaluate its impact. This work provides a foundation for developing training design recommendations.

## Introduction

### Background

Higher education institutions (HEIs) can be effective allies in the fight for diversity, inclusion, and gender equality in the education context and in society as a whole. The leadership, academic and administrative staff, and students of HEIs are increasingly mobilized by the United Nations 2030 Agenda for Sustainable Development Goals [1]. The Higher Education Sustainability Initiative [2] recommends that all formal education curricula should feature education for sustainable development. The principles of gender equality are integral to the goals, targets, and indicators of all sustainable development goals and goal 5, “Achieve gender equality and empower all women and girls*,*” is of particular importance. Regrettably, HEIs continue to be organizations that are both gendered and gendering [3]. Gender equality at HEIs is persistently hindered by structural, institutional, and cultural barriers [4].

Recent statistical reports on gender equality data in the European Union show that female students make up most of the undergraduate population in HEIs, yet several published reports highlight ongoing cultural, institutional, and structural barriers inhibiting gender equality from true realization in this sector [5]. Male students remain a majority among postgraduates [5]. In addition, research highlights the gender inequality that exists in student leadership, demonstrating that although women made up 55% of the student body, they represented only 33% of the leadership in student organizations [6].

In Ireland, the undergraduate student population is comprised of approximately equal numbers of women and men. However, in 2016, the Higher Education Authority [7] reported that most of the country’s student unions’ officers tended to be young, White, and male, an inequality also evident internationally. This gender imbalance has been replicated in senior leadership in tertiary institutions [7]). While much has been achieved since 2016, the subsequent report maintains the recommendation to embed gender equality, and equality more broadly, into teaching, learning, research, and quality assurance processes [8].

The assertion that education and training foster durable change is supported by a substantial body of research emphasizing the transformative potential in both individual and societal contexts. The concept of critical pedagogy by Freire [9] underscores education as a means for empowering individuals to challenge existing inequalities and contribute to social transformation. The United Nations Educational, Scientific and Cultural Organization [1] identifies education as a cornerstone for achieving sustainable development, emphasizing its role in fostering long-term changes in health, gender equality, and environmental sustainability. The European Institute for Gender Equality (EIGE) argues that gender equality training should empower participants to define gender equality principles, identify inequalities, incorporate gender into planning, monitor progress, and assess work from a gender perspective [10]. EIGE defines gender equality training as “A tool and strategy to develop awareness, knowledge, and skills among individuals and to influence organizational processes to promote gender equality and tackle gender-based discrimination” [10].

Coe et al [11] advocate for the integration of equity, diversity, and inclusion training into medical curricula to embed diversity and inclusion as foundational institutional and cultural values. Connolly et al [12] highlight the necessity of training to raise awareness and develop competencies in student leaders, enabling them to proactively tackle gender inequality. These authors assert that such training will have a broader impact on the entire HEI community. Acai et al [13] argue that change must start early in a student’s academic career to address and combat gender inequality in the higher education setting.

Preliminary literature reviews [14-16] suggest that gender equality-based training is being conducted in secondary and tertiary education settings, which includes didactic teaching, face-to-face collaboration projects, site visits, case studies, and coaching. To date, a comprehensive review collating and synthesizing the available evidence on gender equality training for tertiary students has not yet been carried out.

Scoping reviews are designed to examine the scope and limits of knowledge within an emerging field. The scoping review approach allows identification and synthesis of all relevant literature regardless of study design and is useful in clarifying key concepts and definitions in the literature. As detailed in our published protocol [17] we adopt the theory of change (ToC) as the theoretical framework to guide the analysis and interpretation of our findings [18]. The ToC provides a deliberate process for analyzing and outlining how an intervention is likely to be effective, who will benefit, in what ways, and the conditions necessary for its success.

The United Nation women’s approach to gender equality training is grounded in the ToC recognizing that achieving gender equality requires a long-term institutional commitment, in addition to the development of key competencies for individuals [19]. ToC offers a conceptual foundation for how gender equality can be achieved through training, by emphasizing the interconnected roles of knowledge, desire, and ability as the necessary components for lasting change. It also aligns with the broader goal of embedding gender equality into institutional and cultural norms.

### Objectives

Our work aimed to support gender equality in HEIs by working with the leaders of the future (men, women, and nonbinary) to address biases and accelerate culture change. This review is a component of a detailed needs assessment following the Kern’s 6-step framework for curriculum development and implementation [20]. Understanding the nature of interventions and approaches currently being used to improve the knowledge, skills, and attitudes of students is an integral step in the design of an effective training program. The findings of this scoping review will inform education researchers, faculty, and academic administrators on the application of gender equality training, pinpoint gaps in the literature, and help identify opportunities for instructional designers and subject matter experts to improve course content.

The primary objectives of this scoping review were to produce a descriptive overview of gender equality training and interventions for students in higher education or postsecondary education, which will inform curriculum development for skills training in the domain of gender equity. The secondary objectives were (1) to determine the methodology and content of gender equality training delivered to students; (2) to establish the skills and competences that are required by students to promote gender equality; (3) to ascertain how gender equality training is evaluated; (4) to review the extent to which the concept of leadership is included in gender equality interventions; and (5) to establish how gender equality leadership skills are fostered among students.

## Methods

### Overview

The review was structured using the 6-stage framework developed by Arksey and O’Malley [21], as follows: (1) identifying the research question; (2) identifying relevant studies; (3) study selection; (4) charting the data; (5) collating, summarizing, and reporting the results; and (6) expert consultation. This approach was chosen due to its well-established rigor and effectiveness [22]. Our review protocol has been peer reviewed to ensure the appropriateness and effectiveness of our methods [17].

This review involved the analysis of publicly available empirical research and the production of secondary data; therefore, ethics approval was not required.

### Identifying the Research Question

Using the population, concept, context framework [23], the primary and secondary research questions were developed (Textbox 1) in alignment with the previously stated objectives. The population included students in higher education; the concept, gender equality training; and the context encompassed all HEIs.

**Primary and secondary research questions that guide this scoping review.**

**Primary**

- What is the current nature and scope of gender equality training and interventions delivered to students in higher education?

**Secondary**

- How is gender equality training delivered to students, and what are the key topics included in the intervention?
- What specific skills and competences are taught to students to enable them to promote gender equality?
- How is gender equality training evaluated? To what extent is the concept of leadership included in gender equality interventions?
- How are gender equality leadership skills fostered among students?

### Identifying Relevant Studies

Following a preliminary search to identify key terms, the search strategy was designed in consultation with an experienced research librarian. The search terms comprised 3 thematic combinations, including: (1) gender equality training, (2) HEIs, and (3) students, each separated with the Boolean operator AND. Within each thematic combination, search terms were separated using the Boolean operator OR. Wildcards were used to ensure the inclusion of plurals and variation in spelling across the search terms. The search was limited to studies and other sources published between January 2011 and November 2021.

An example of the search strategy used in 2 of the key databases is shown in Textbox 2.

**Search strategy showing the search string for the APA, Psycinfo, and CINAHL databases.**

1. TI (gender N2 training OR bias N2 training OR discrimination N2 training OR diversity N2 training OR equality N2 training OR inclusion N2 training OR sexuality N2 training) OR AB (gender N2 training OR bias N2 training OR discrimination N2 training OR diversity N2 training OR equality N2 training OR inclusion N2 training OR sexuality N2 training)
2. TI (gender N2 course$ OR bias N2 course$ OR discrimination N2 course$ OR diversity N2 course$ OR equality N2 course$ OR inclusion N2 course$ OR sexuality N2 course$) OR AB (gender N2 course$ OR bias N2 course$ OR discrimination N2 course$ OR diversity N2 course$ OR equality N2 course$ OR inclusion N2 course$ OR sexuality N2 course$)
3. TI (gender N1 program* or bias N1 program* or discrimination N1 program* OR diversity N1 program* or equality N1 program* OR inclusion N1 program* OR sexuality N1 program*) OR AB (gender N1 program* OR bias N1 program* OR discrimination N1 program* OR diversity N1 program* OR equality N1 program* OR inclusion N1 program* OR sexuality N1 program*)
4. TI (gender N1 awareness* or gender N1 bias or gender N1 equality OR gender N1 inclusion OR gender N1 equity OR sex N1 bias) OR AB (gender N1 awareness* or gender N1 bias or gender N1 equality OR gender N1 inclusion OR gender N1 equity OR sex N1 bias)
5. 1 OR 2 OR 3 OR 4
6. TI (“higher education” OR “third level education” OR “tertiary education” OR university* OR college$) OR AB (“higher education” OR “third level education” OR “tertiary education” OR university* OR college$)
7. TI (Undergraduate$ OR postgraduate$ OR student$) OR AB (Undergraduate$ OR postgraduate$ OR student$)
8. 1 AND 2 AND 3
9. LIMIT 8 to 2010-2021

Systematic searches were carried out by the librarian in 6 databases of peer-reviewed research, including APA PsycInfo, CINAHL, Embase, MEDLINE (Ovid), Scopus, and Web of Science, and 3 additional databases, MedEdPortal, MedEdPublish, and Open Grey, to identify any gray literature that could further inform the review. The search was limited to titles, abstracts, and key words, to optimize the return of articles and sources of evidence with an appropriate focus on the topic in hand. All identified citations were collated in EndNote 20.2.1 (Clarivate Analytics) and duplicates were removed. This EndNote library was exported to Rayyan (Rayyan Systems, Inc) [24] to facilitate collaborative evidence screening.

### Study Selection

In line with the recommendations from Peters et al [23], evidence selection was based exclusively on agreed eligibility criteria. These inclusion and exclusion criteria were developed in accordance with the previously stated research questions, building on the elements of the population, concept, context framework (Textbox 3). A reflexive approach was used throughout the selection process to adapt and develop the eligibility criteria in an iterative fashion through discussion within the research team throughout the course of the screening process.

Using Rayyan, CC and MP screened the titles of all sources independently and disagreements were resolved through discussion. The included abstracts were then screened by CC and MP in a similar fashion. The full-text studies were retrieved and CC, MP, SL, PH and TD collectively reviewed the first 10 texts to pilot the eligibility criteria framework. Group discussion was used to further clarify and develop the criteria. The remaining texts were then reviewed by 2 reviewers independently, with any disagreements between these reviewers resolved by discussion. CC and MP acted as third reviewers if a consensus could not be reached.

The reference lists of the included texts were back searched by MM and CC for further relevant studies and sources. Title and abstract screening by MM and CC of articles from all issues of 2 key journals, *Gender, Work & Organization*, and the *Journal of Gender Studies*, published between January 2011 and November 2021, did not yield any additional studies which met our inclusion criteria.

**Eligibility criteria for evidence selection.**

**Inclusion criteria**

- Population: undergraduate or postgraduate students in higher education
- Concept:
  interventions (eg, campaign or workshop) promoting gender equality awareness or gender equality competences
  intervention that includes a specific goal or objective to educate or raise awareness of gender equality or to foster competence in gender equality
  interventions inside or outside of the academic curriculum, for example, project within a module or exercise within a module, or course or program external to course of study
  articles or sources focusing on the experience of participation in an intervention
  interventions can also focus on other aspects of equity, diversity, and inclusion training, provided gender equality is included
  interventions involving gender equality in terms of gender diversity (transgender, nonbinary, and gender diverse students)
  training in gender equality for student teachers
- Context: higher education institutions
- Publication dates: between January 2011 and November 2021
- Sources:
  peer-reviewed literature and gray literature (dissertations, websites, conference papers, corporate documents, government reports, preprints, proceedings, research reports, and periodicals)
  secondary research, that is, literature, systematic or scoping reviews
- Language: studies in English

**Exclusion criteria**

- Population: primary or secondary students, higher education staff, and general public
- Concept:
  measuring gender balance in higher education courses
  strategies to promote equality among applicants to courses
  experiences of gender or gender inequality among students
  impact of gender on subject-specific competences, for example, programming or spatial awareness, among others
  recruitment or retention of female students in academic courses
  gender equality in health care
  mentorship programs
  diversity or equity, diversity, and inclusion programs that do not include a gender aspect
  gender studies modules in which the goal is to inform on a variety of gender-related theories
  articles focusing on a whole-of-campus institutional change
  research on students’ attitudes to gender equality unless these form part of a specified intervention
  exploring the perceptions or experiences of people delivering the intervention
- Context: primary or secondary schools, youth services, and community services
- Publication dates: before January 2011
- Sources: books and book and film reviews
- Language: studies for which no English translation is available

### Charting the Data

A standardized data charting tool (Textbox 4) was developed to extract data. This tool was adapted by the research team from the Joanna Briggs Institute template data extraction instrument [23] to align with the review objectives and questions.

**Data charting form to collect extra data and chart data for each paper.**

**Heading**

- Study details (authors, year, title, and citation)
- Location
- Context
- Type of source
- Study aims or intervention aims
- Participants details and sample size
- Research questions addressed
- Study design
- Intervention style and duration
- Intervention content
- Intervention methods
- Outcome evaluation measures
- Key findings

**Miscellaneous**

- Interesting observations
- Gender equality theories employed
- Incorporation of gender equality and leadership
- Incorporation of intersectionality
- Incorporation of transgender and gender diverse inclusivity

The charting form was expanded and refined in an iterative fashion by all authors during the full-text screening process. Data extraction was carried out by 2 reviewers working independently, with a single finalized form for each study agreed through collaborative discussion and communication.

## Results

### Collating, Summarizing, and Reporting the Results

**Figure 1:**
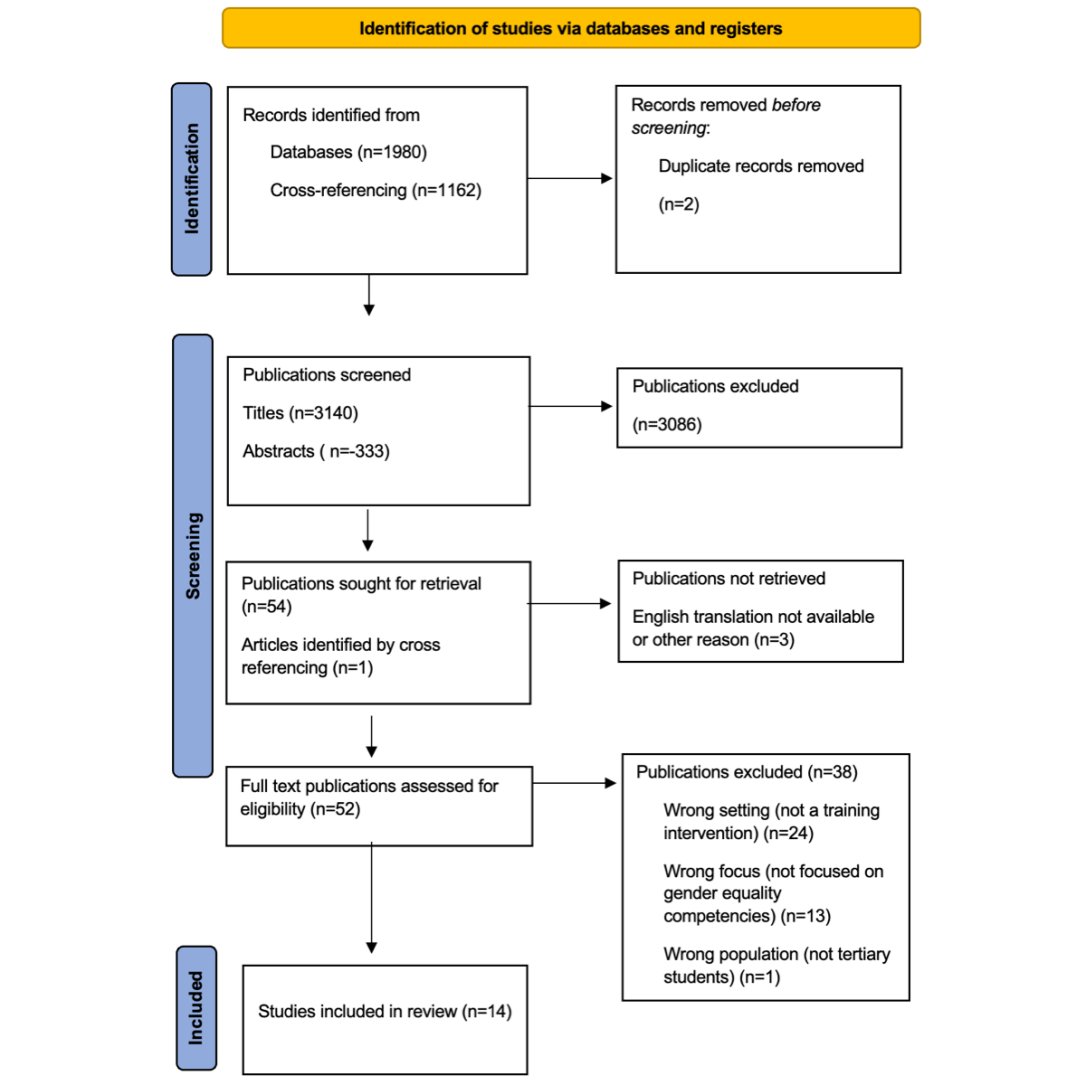
PRISMA-ScR (Preferred Reporting Items for Systematic Reviews and Meta-Analyses Extension for Scoping Reviews) diagram detailing the process for identification and selection of articles included in this study.

The study selection process is presented in a flow diagram (Figure 1) as per the PRISMA-ScR (Preferred Reporting Items for Systematic Reviews and Meta-Analyses Extension for Scoping Reviews) in Multimedia Appendix 1 [25]. On the basis of the database searches, and the subsequent hand search of other sources as described in the Identifying Relevant Studies section, 3142 titles were screened. This yielded 333 abstracts for further screening. In total, 14 full-text articles were selected for inclusion in the review.

### Study Characteristics

Basic descriptive analysis is the most suitable approach to data analysis given the exploratory nature of a scoping review [23]. In total, 13 of the publications included in the final sample were peer-reviewed academic articles and 1 was a conference paper. The details of each article, including the country in which the study took place, the participants, aims, methodology, evaluation, and outcomes, are summarized in Table 1. Much (5/14, 36%) of the work in this field has been conducted in the United States and in Europe, with Spain (3/14, 21%) and Sweden (1/14, 7%) contributing. A further 3 (21%) studies were conducted in Turkey, with a single study located in each of Taiwan (1/14, 7%) and South Africa (1/14, 7%). In keeping with the agreed search strategy for this review, the included studies were published from 2011 to 2021. The published studies demonstrate a growing interest in the topic, as 57% (8/14) of the studies were published in the latter 3 years of the selected period (2019 to 2021).

**Table 1:** Summary of included studies.

Study	Country	Population	Aims	Methodology	Evaluation	Outcomes
Shields et al [26], 2011	United States	N=118; female: n=62, 52.5%; male: n=53, 44.9%; nonresponders: n=3, 2.5%; mean age: 19 y	To evaluate game-like simulation in teaching the nature and consequences of unconscious biases and stereotyping, which underlie gender inequity.	Experimental group divided into 2 teams, who participated in WAGES^a^–Academic, a game that simulates academic career progression with and without advantages. Separate control group played Chutes and Ladders; 3 phase study; pretest evaluations, immediate and delayed posttest evaluations; Kolb’s experiential learning model used [27].	Neosexism scale at pretest; KGE^b^ scale at all 3 stages; single open-ended question in final evaluation, “Since you played the game, have you thought about issues or made observations that you might not have before? If so, what are they?”	No difference in sexism scores among WAGES–Academic participants; no difference in KGE scores at baseline. Significant increase in KGE scores for intervention (both teams) versus control. KGE scores remained higher for intervention group at third evaluation; 24 participants indicated that they had thought about or made observations related to issues raised by WAGES–Academic; 15 noted gender bias, 5 wanted to learn more, 4 were unsure.
Kennedy et al [28], 2011	Turkey	N=546 students taking 2 introductory courses in Sociology and Psychology; gender: not reported; age: not reported	To assess the merit of showing movies as critical pedagogy; to develop student interest in serious social issues, including gender equality, and encourage critical agency in society.	In total, 3 cohorts of students watched 3 different movies over 3 successive academic terms; no comparator or control group; postmovie discussion undertaken	Postevent analysis of 3 student essays completed after watching the movies; 14-item survey completed by 112 students	Student essays indicated a development of consciousness in 3 critical subject matters, the death penalty, gender inequality and prejudice. Students rated the utility of watching movies favorably concluded that “movies help the students build critical perspectives on the ‘social’ through interest development on sociological topics;” 105 students reported development of a high level of critical perspective or a critical perspective; 7 students indicated that they had not developed a critical perspective.
Falk et al [29], 2012	Sweden	N=9 students participated in a gender module; gender: not reported; age: not reported	To evaluate Euro-Education: Employability for all, a multi-center project in which 4 course modules relating to different aspects of employability were developed: gender, age, disability, and ethnicity.	The module consisted of tutorial groups, lectures, tasks, and seminars. Students used a variety of electronic learning applications. Activities included a critical review of the labor market, an “exchange” in a university Occupational Therapy Department, and development of a “course” to address gender impact as the final assignment.	Continuous evaluation by students and teachers as part of feedback process throughout; postcourse oral feedback; questionnaire; overall assessment by EU^c^ Commission (method not stated)	Students’ evaluation was positive about increasing awareness and knowledge of gender theory, its application to employability, and development of new skills; technical aspect was challenging at times; teachers recommended precourse training on the electronic learning applications; the project received a global score of 8/10 from the EU Commission.
Case et al [30], 2014	United States	Study 1: n=177, 80% female; study 2: n=131, 71% female	To examine the effectiveness of 2 interventions (privilege list handout and testimonial video), coupled with reflective writing, in raising awareness of heterosexual (study 1) and male (study 2) privilege.	Study 1: participants assigned to control and 2 comparator groups, who received either a privilege list or watched a video); study 2: same method with different content	Study 1: pre- and postsurvey with 3 scales (heterosexual privilege awareness, internal and external motivation to respond without prejudice) and a reflective summary; study 2: pre- and postsurvey with 5 scales (male privilege awareness, modern sexism, hostile and benevolent sexism, and the motivation scales as above) and reflective summary	Study 1: Neither intervention had a more significant impact than the other. The authors indicated that both techniques were helpful in enhancing learning about heterosexual privilege; study 2: The video intervention increased male privilege awareness, but the handout did not. The authors concluded that further research is required to target aspects of sexism which neither intervention addressed.
Abrams et al [31], 2016	United States	N=13; male undergraduate (n=10, 77%) and graduate (n=3, 23%) engineering students invited to participate. One withdrew, one deemed not suitable, one went on placement for second semester; 10 students participated in full program	To evaluate the AWE^d^ program, which aims to improve women’s retention in engineering fields by promoting allyship among student population. Content focused on gender equity, implicit bias, micro aggressions, and sociocultural conversations.	Participants completed the 1-y AWE program consisting of initial training (workshops, seminars, and speakers) over 4 d; up to 5 hr. per wk. in semester 1 spent on reflection, further training sessions and planning outreach activities, which were then delivered to 447 students and staff on campus; Broido’s Model of Allyship was used as framework for training design [32].	Authors intended to assess using a mixed methods approach, including qualitative and quantitative techniques; pre- and postsurveys, focus groups and interviews; this paper presents the results of the presurvey and 2 free-text response questions	After the initial 4 d training, most participants agreed that they could identify and explain microaggressions, knew how to address bias and discrimination, and were comfortable to do so; participants gained insight, awareness, confidence, and new perspectives from attending the training; participants felt they would change their behaviors, have increased awareness, have more confidence to speak up, become more inclusive daily, and become a role model for their peers.
Freedman et al [33], 2018	United States	N=143 college students; female: n=65, 45.4%; male: 77, 53.8%; nonresponder: 1, 0.7%; mean age: 20 y (a second study involved high-school students, which is not reported here)	To examine the impact of experiencing an “aha” moment about assumptions about women in science on subsequent attitudes toward women in science.	Randomized controlled trial in which participants play a logic-puzzle game that is won by making a realization about a character. In the control game, the character is a professor; in the intervention game, the character (a scientist) is a woman. Numbers of participants in each group not reported.	Questionnaire included a monetary allocation task in which participants had to allocate US $500 among 14 college organizations, 2 of which supported women in STEM^e^; shortened version of the Attitudes toward Women in Science Scale; the ambivalent sexism inventory	Most students who used gendered pronouns assumed that nongendered scientist characters were men; contrary to hypotheses, the intervention condition did not increase positive attitudes toward women in science, decrease sexism, or increase donations to women in STEM organizations.
Altınova et al [34], 2016	Turkey	N=65 social-work students; female: n=39, 65%; 26 male: n=26, 40%); age: not reported	To examine the impact of the HREP^f^ on improving the gender perceptions. The HREP aims to raise social consciousness concerning human rights violations that women encounter.	Pretest posttest design with experimental group (n=32) participating in HREP and control group (n=33) who did not participate; participants engaged in narration, role play, case study, and problem-based learning during 12 two-h training sessions.	25-item Gender Perception Scale	GP^g^ levels of the experimental group were increased; no change in GP levels for control group; concluded that the HREP is effective
Segovia-Pérez et al [16], 2019	Spain	N=50 female students from a variety of academic backgrounds over 2 consecutive years with 25 students in each cohort; mean age: 22 y	To evaluate a women’s leadership program for university students which sought to ensure acquisition of skills, competencies, and tools for leadership by the participants, in addition to increased self-confidence.	The program involved 24 h of classes, a case study, visits to companies, and the European Parliament, and a coaching system. Subjects included personal branding, communication, networking, public speaking, negotiation, leadership techniques, and business management; the second cohort also completed a leadership test.	A self-administered survey with 9 questions using a 10-point Likert scale related to the program, prior knowledge, potential future impact, and a global evaluation; 3 focus groups with 8 participants in each group; 4 individual informal interviews	The participants rated the course highly, with scores of 8 out of 10 in all areas of the survey, other than prior knowledge; the qualitative data indicated an improvement in specific tools and skills, and a positive change in attitudes and self-confidence of the participants.
Toraman and Özen [14], 2019	Turkey	N=433 students in the Faculty of Education; female: n=319, 73.7%; male: n=144, 3.2%; age: not reported	To determine the opinions of the participants regarding gender equality, and to compare their opinions before and after taking a compulsory gender equality course; later, described third goal to explore association with participant variables.	Students participated in a training course over one semester, which included the following topics; the concept of gender, sociology of gender, gender and family, gender and religion, gender and language, gender and media, gender and body images, gender, work life and labor, feminist movements, and social change. Limited description of learning and teaching activities.	Descriptive study with pre- and postintervention administration of the Gender Equality Scale, with validation of the scale for university students included in the methods; data were also collected on background participant variables; logistic regression used to assess the extent to which background variables affected participants’ opinions	Initially, most participants did not believe that men were superior to women. Unexpectedly, after the course, participants were more likely to develop the opinion that men are superior to women, whereas there was no impact on opinions relating to women being dependent on men; men were more likely to believe that they are superior, and that women are dependent on men; significant variables in the regression analysis included academic background, father’s education status, newspaper reading by family members, and location of family home.
Gorrotxategi et al [35], 2020	Spain	N=64 social education students; female: n=52, 81%; male: 11, 17%; and 1 did not identify themselves as male or female; mean age: 20 y	To measure the students’ knowledge about transgender people, and the attitudes of students toward gender and transgender people, before and after an education program.	Training was based on the “Creative Factory” intervention model and consisted of a weekly training session on gender and transgender learning over a 4-mo period. The goal of the Creative Factory model is to “enable students...to analyze social realities to generate discussion and innovate ideas to design successful practices.”	Pre- and postintervention questionnaires; 12-item short version of the Gender and Transphobia Scale; transgender knowledge assessed with single item scale	Level of transgender knowledge was increased by the intervention (*P*<.05); improvements were noted in gender bashing and transphobia dimensions but these were not statistically significant; the data suggests that men had higher levels of gender bashing attitudes (*P*<.05) and transphobia, but this latter difference was not statistically significant.
Liao and Wang [36], 2020	Taiwan	N=82 medical students; gender: not reported; age: not reported	To investigate whether the integration of the gender perspective into literature studies would create any difference among students in gender awareness and critical thinking.	Intervention entailed twice weekly literature study sessions over 15 wk, self-study, electronic discussion between both groups. Experimental group received gender perspective training and were introduced to gender-related terms to facilitate discussion. Through literature, this group were encouraged to consciously reflect on traditional and socially constructed gender norms.	Quasi-experimental study with a control group (n=41) and experimental group (n=41); both groups completed the CTDA^h^ and the Chinese version of the Gender Awareness Scale pre- and postintervention	With respect to gender awareness, the findings show that following the integration of the gender perspective into literature studies, medical university students had significantly higher posttest scores for “public gender consciousness” and “private gender consciousness;” regarding critical thinking, they also had significantly better posttest scores in “systematicity and analyticity,” “maturity and skepticism,” and “inquisitiveness and conversance.”
Locke et al [37], 2021	United States	Male undergraduate and graduate STEM students; this paper includes the reflections of a male engineering student ally, and a research adviser	Update of Abrams et al [31]; the success of the AWE program led to the development of a leadership course that has been offered in the College of Engineering every semester since autumn 2016.	A total of 14-week course incorporating videos, workshops, case studies, and group discussions; participants engaged in: information gathering to develop awareness of gender equity challenges in engineering; meaning making to examine personal biases; and, contextual application of strategies that promote inclusive engineering climates.	Pre- and postcourse survey with Likert-type scales	Increase in self-reported efficacy of participants; this course helped the male ally to “develop the skills and mindset needed to make...difficult conversations productive.” It gave him “the tools needed to identify the various kinds of situations that contribute to a hostile environment and how to better diffuse them;” created “an enhanced supportive environment” in a research laboratory, as reported by a female research adviser.
Bosch et al [38], 2021	Spain	N=19 final year students taking a sociology of gender course female: 15, 79%; male: 4, 21%; age: not reported	To evaluate a SL^i^ project, in which students deliver workshops in high schools on gender and technology. The optional project within the sociology of gender course was intended to enhance student understanding of topics covered in the course.	For each workshop, students created a presentation with interactive activities, and showed a video. Their assessment consisted of an oral presentation to the rest of their university class and a self-reflection; 13 workshops were carried out over 4 y.	Qualitative analysis of 16 university student self-assessment reports; quantitative survey data from high-school students (n=284) and teachers (n=13) on a range of items, such as usefulness, alignment with student need, evaluation of pedagogical tools, opportunities for participation and a global evaluation. This quantitative data are not relevant for this review but the abovementioned description is included for completeness.	All students were satisfied with the experience and the course grades for these students were higher than the class average. The authors felt that these students went “far beyond the curriculum” and “reached a deep understanding of the multiple dimensions on the topics of gender and technology.” Other themes which emerged included social transformation through SL, acquisition of competences, such as improved communication, putting empathy into practice, empowerment and “professionalization” of the participants.
de Villiers et al [39], 2021	South Africa	N=15 “student leaders” from various faculties; all male; age range: 20-25 y	To evaluate the OMC^j^ intervention for university settings. OMC aims to encourage development of equitable relationships between men and women, thereby preventing gender-based violence and HIV transmission.	A case study design describing the adaptation and implementation of OMC, in which 5 participatory workshops were conducted. Content was related to personal values, belief systems, societal gender-based norms, rape and consensual sex, courage and bystander intervention, and healthy relationships.	Qualitative data collection methods, including pre- and postintervention focus groups, discussion content and researchers’ field notes documented during the intervention workshops, participant reflections were collected after each workshop via open-ended questions and 5 semistructured interviews were conducted 6 mo after the intervention; thematic data analysis was used.	The data demonstrated “increasing accountability” on the part of the male student leaders to prevent sexual violence. The authors note that critical engagement and dialogue on sexual violence is shown to shift key norms on gender equality, on being a man and reflection on their role in preventing sexual violence; this work resulted in the development of the Men with Conscience intervention; a 6-h adapted intervention based on OMC.

^a^WAGES: Workshop Activity for Gender Equity Simulation.

^b^KGE: knowledge of gender equity.

^c^EU: European Union.

^d^AWE: allies for women engineers.

^e^STEM: science, technology, engineering, and mathematics.

^f^HREP: Human Rights Education Program for Women.

^g^GP: gender perception.

^h^CTDA: Critical Thinking Disposition Assessment.

^i^SL: service-learning.

^j^OMC: One Man Can.

### Settings

All these educational programs (N=14) took place at individual tertiary academic institutions. One involved collaboration with secondary schools within the same country requiring participants to present to school students, 2 programs incorporated workplace visits, and 1 used an online discussion forum. Most studies did not provide information on how the program was financially supported. Some of the training interventions were undertaken as official curricula to which the participants were enrolled as part of their required studies. Most studies recruited volunteers, one via a competitive recruitment process and another accepted nomination from teachers. One study reported that learners were incentivized to participate in the training experience with a US $300 book token per term [31].

### Participants

All studies provided some demographic information on participants and the number of participants in the studies ranged from 9 to 546, with an average of 134 participants. With respect to gender of the participants, some of the interventions specifically targeted either women [16] or men [31,37,39]. In the remainder of the studies which reported participant gender (7/14, 50%), the majority were female. When reported, the age range was typical for higher education as most participants were in their early twenties. Most studies involved participants from single faculties. There was a slight predilection for interventions involving students in education and the social science programs [14,28,34,35,38]. There was also representation from health sciences, including medicine [36] nursing [39] psychology [26,30] and occupational therapy [29], in addition to engineering faculties [31,37]. In 2 (14%) studies, the participants were from a variety of academic programs: Business, Social Sciences, Law, Engineering, and Architecture [16,37]. Freedman et al [33] recruited participants from student accommodation. One study did not provide information on participant specialty.

### Content

The works reviewed were from countries with considerable variation in political-cultural contexts, civil liberties, and personal freedoms and thus the content of the courses was widely varied reflecting local needs.

The language used to describe content themes was also disparate. The main content areas covered in the training programs are listed in Table 2. In total, 5 (36%) of the studies from this heterogeneous group explored the impact of courses, modules, or programs, which directly aimed to increase awareness of gender and gender-based issues or enhance gender equality knowledge [14,29,34,35,37]. Another group of studies sought to enhance knowledge and skills indirectly through another medium, such as movies [28], testimonial videos [30], games [26,33], and literature studies [36]. In addition, 2 (14%) studies reported on the same initiative, an allyship program in a college of engineering that encompassed gender equity issues [31]. A subsequent paper [37] described the extension of the initial project. A single study was identified which related specifically to a leadership program for women [16]. The final study related to a service-learning project on gender and technology [38].

**Table 2:** Content themes identified and the frequencies of inclusion in the individual curricula from studies examined in this study (N=14).

Theme	Frequency, n (%)
Allyship	2 (14)
Gender awareness	2 (14)
Gender barriers	1 (7)
Gender equality	5 (36)
Implicit bias	5 (36)
Intersectionality	1 (7)
LGBT^a^ marginalization	2 (14)
Male privilege	1 (7)
Microaggressions	1 (7)
Stereotypes	1 (7)

^a^LGBT: lesbian, gay, bisexual, and transgender.

### Gender Equality Leadership Skills

The concept of leadership featured explicitly in 2 (14%) of the included articles [16,37]. Both publications detailed how gender equality leadership skills were fostered among the participants. The concepts of social identity, privilege, microaggressions, and implicit bias were explored by Locke et al [37] in a leadership program for male student allies. In contrast, Segovia-Pérez et al [16] ran a leadership program for female students which covered topics such as personal branding, communication, networking, public speaking, negotiation, leadership techniques, and business management.

### Study Design: Inputs and Activities

The most common (4/14, 29%) study type was an interventional mixed methods pre-post design, wherein a single group of participants completed self-assessments of confidence, ability, and knowledge before the intervention and then participated in a module or workshops. The learners completed the same assessments after the intervention in addition to participating in focus groups or interviews [14,26,31,35]. Interventional studies using pre- and postintervention questionnaires were the next most common (3/14, 21%) study design [16,30,37]. Moreover, 2 (14%) studies used a quasi-experimental design [36,39], and 1 (7%) used a randomized controlled trial design [33]. The final pool of articles comprised 5 (36%) qualitative studies using after event analysis only ranging from educators’ reflections (2/14, 14%) to analysis of student diaries or essays (2/14, 14%) [28,38] and an after event questionnaire with oral feedback (1/14, 7%) [29].

### Delivery Methods

Methods of delivery varied widely. Most studies (11/14, 79%) were described as longitudinal events that occurred over multiple sessions, ranging from several months to a year. One study consisted of 15 European Credit Transfer and Accumulation System (ECTS) where 1 ECTS is equal to between 25 and 30 hours. In addition, 2 (14%) studies described single event game base interventions. The format of teaching was also wide-ranging and included lectures and workshops, guest speakers, small group discussions, workplace visits, role play, videos, movies and literature analysis, student presentations, and online discussion.

### Assessment and Evaluation: Outcomes

Most studies reported no assessments of learner competency after training. Most studies (8/14, 57%) measured learner outcomes via survey instruments examining perceptions, sympathies, or confidence. In total, 2 (14%) studies assessed outcomes with participant focus groups. Moreover, 2 (14%) studies measured learning by thematically analyzing reflection writing or diaries. No study reported on transfer of learning.

Significant increase in knowledge of gender equity scores [26], gender perception scores [34], and higher posttest scores for “public gender consciousness” and “private gender consciousness” [36] were demonstrated for intervention participants versus control.

Students self-reported the development of critical perspective [28], increased male privilege awareness [30], increased awareness and knowledge of gender theory [29], and increased level of transgender knowledge [35]. Students agreed that they could identify and explain microaggressions and knew how to address bias and discrimination [31].

In qualitative studies, data from focus groups with participants following training indicated “increasing accountability” on the part of the male student leaders to prevent sexual violence [39], and a positive change in attitudes and self-confidence [16].

Unexpectedly, participants from a course in the faculty of education in a Turkish university were more likely to hold the opinion that men are superior to women postintervention and there was no impact demonstrated on opinions relating to women being dependent on men. Regression analysis included academic background, father’s education status, newspaper reading by family members, and location of family home [14]. In addition, contrary to the study hypotheses, a logic-puzzle game intervention from the United States did not increase positive attitudes toward women in science, decrease sexism, or increase in-game donations to women in science, technology, engineering, and mathematics organization [33].

### Theoretical and Conceptual Foundations

The array of theoretical and conceptual foundations cited by the authors is displayed in Table 3. Most studies (12/14, 86%) introduced and discussed theoretical underpinnings as a reason to provide training and less so for instructional design and program content. Furthermore, there was little or no reference to educational learning theories or pedagogical approaches when designing learning objectives, delivery methods, or evaluation and assessment of the programs. Exceptions include the use of Broido’s model of allyship [32] by Abrams et al [31] and Kolb’s experiential learning model [27] by Shields et al [26].

**Table 3:** Underpinning theories.

Study	Gender equality theories used
Shields et al [26], 2011	Work-family balance, salary, mentoring, workplace climate, and token status
Kennedy et al [28], 2011	Critical teaching
Falk et al [29], 2012	Gender theory and gender as a social construction
Case et al [30], 2014	Privilege, modern sexism, hostile and benevolent sexism, and prejudice
Abrams et al [31], 2016	Gender equity, implicit bias, micro aggression, and sociocultural conversations
Altınova et al [34], 2019	Gender equality from a human rights perspective
Freedman, et al [33], 2020	Gender stereotypes; implicit and explicit gender bias; social psychology—challenging the assumption of invulnerability to bias
Segovia-Pérez et al [16], 2019	Stereotypes and social role theory. Inclusive and equitable quality education, gender equality, and empowerment
Toraman and Özen [14], 2019	Social cognitive theory; gender and its inequalities originate from learned or taught behaviors
Gorrotxategi et al [35], 2020	Gender bashing, transphobia, and genderism
Liao and Wang [36], 2020	Socially constructed gender norm, patriarchal system, gender politics, and ideology
de Villiers et al [39], 2021	No explicit gender equality theories

## Discussion

### Principal Findings

This scoping review explored and mapped the evidence related to gender equality training and interventions for university students using a ToC lens. We described inputs and activities in terms of content areas, delivery modes, participants, and settings. We described outcomes and impact in terms of evaluation and assessment.

It was not possible to measure the long-term systemic impacts of the interventions due to limited postintervention follow-up and the absence of transfer of learning assessments. The studies collectively indicate potential for improved gender equity knowledge and leadership skills in diverse educational contexts and increased capacity to address microaggressions, implicit bias, and gender-based discrimination. However, it is important to note that self-reported data are typically considered low-quality evidence for evaluating the effectiveness of teaching interventions [40], especially if the questions focus primarily on learner satisfaction rather than assessing actual knowledge acquisition or behavioral change. Improvement or impact on skills to support gender equality is less frequently observed or demonstrated as an outcome in the literature we reviewed, as is any change at the organization level. Critical assumptions underpinning these studies included the willingness of participants to engage actively with the material and the alignment of local cultural contexts with course content.

The study by Toraman and Özen [14] highlights the unintended reinforcement of gender stereotypes, emphasizing the impact of cultural and political contexts. Similarly, the adverse effects of the Freedman et al [33] training intervention on explicit attitudes toward women in science serve as a cautionary tale, highlighting the importance of educational research in designing interventions that reduce biases without provoking defensiveness.

The usefulness of the studies we identified to inform curriculum design and provide actionable insights for educators is constrained by a lack of theoretical grounding, limited long-term follow-up, and reliance on self-reported data. This aligns with the findings of Guthridge et al [41] who, in their systematic review of interventions, aimed at enhancing gender equality and questioned whether the interventions they examined effectively led to substantive change. The EIGE understands developing competence in gender equality as being able to identify and change gender stereotypes and gendered roles [10]. Thus, gender equality training should enable and empower participants to (1) define and understand gender equality principles, (2) identify gender inequalities in their field, (3) incorporate gender in their planning and policy implementation, (4) monitor progress, and (5) review and assess their work from a gender perspective [10]. We have incorporated these concepts with developments from the wider gender equality training literature in subsequent sections and used a ToC to provide a lens through which we can consider and synthesize our findings. From this synthesis, combined with our own experience as educators and consultation with experts in gender equality, we have created recommendations for both practice and future research to help close the gap between the current practice and desired outcomes.

### Instructional Design

Pedagogical frameworks should guide the design and implementation of educational activities. These frameworks are based on established principles of teaching and learning and provide a structured approach to designing effective and engaging learning experiences. Without a solid theoretical foundation, it can be challenging to determine the best instructional strategies to use, how to sequence and structure the content, and how to assess whether learners have achieved the desired learning outcomes. A lack of underpinning theory in the instructional design of training can lead to an inconsistent approach and potentially ineffective training. By grounding an approach in theory, educators aiming to upskill university students in gender equality skills can design training that is more likely to be effective in achieving the EIGE 5 categories of learning outcomes. Interventions to combat biases are particularly effective when they involve active participation rather than passive learning [26,33].

### Diversity of Content

Ensuring diversity of content to cover knowledge, skills, and attitudes is critical for designing effective learning experiences. Not only must learners develop a deeper understanding of the subject matter and its various dimensions, but they must also develop the skills and attitudes to bring about change in behaviors [19]. The terminology used to describe content themes varies widely in the reviewed papers. The key content areas addressed in the training programs include knowledge of gender and gender-based issues, allyship, leadership, and bias. Communication skills, teamwork, and leadership skills training can support learners to become more adaptable and better equipped to promote a gender equality agenda.

### Leadership

Within the body of evidence included in this scoping review, the concept of leadership appears underrepresented in most gender equality training initiatives. One notable exception is the women’s leadership program for female university students described by Segovia-Pérez et al [16]. The authors demonstrated that leadership training enhanced learner self-confidence and their view of their own capacities, providing tools and guidelines for professional communication and personal branding. Enhancing leadership skills were also considered by Locke et al [37] who described a course that focused on gender equity and the practice of inclusive leadership for male allies in the STEM fields. The authors stated that this course would continue to be offered, with plans to explore changes in student behaviors in addition to investigating potential trends or differences in students in varying engineering majors and academic careers stages. Furthermore, a similar workshop for new engineering staff at a prominent state company has also been offered, thereby demonstrating the potential of gender equality training initiatives to promote linkages between higher education and industry, and indeed society more widely.

### Intersectionality

Case et al [30] recommends an intersectional approach for future work: “Future research considering several forms of privilege within the same intervention would provide information about the learning process.” They conclude that “teaching and learning about LGBT psychology through an intersectional lens allows students from a variety of backgrounds to connect seemingly irrelevant systems of oppression and privilege to their own social identities and social locations.” These authors suggest that training which addresses intersections of identity to allow further understanding of sexism, heterosexual privilege, and male privilege will further raise awareness of not only privilege but also the complexities of identity and the matrix of oppression [30]. Gorrotxategi et al [35] demonstrates that interactive training in gender education using the Creative Factory methodology creates a context of reflection and knowledge generation and promotes a significant improvement in knowledge about transgender and slight improvements in transphobia. Bosch et al [38] report on a service-learning intervention, using community engagement pedagogies that facilitate learners to volunteer with an agency and engage in reflection activities to deepen understanding. After the experience in schools with a great diversity of social backgrounds, learners were motivated to reflect on intersectionalities, such as origin or class.

### Delivery, Implementation, and Evaluation

The nature of gender equality training interventions identified for this review are heterogeneous. This can be seen in terms of the format of interventions, and whether gender equality knowledge and skills were directly seen in terms of the population, concepts, and contexts. Delivery, implementation, and evaluation are critical components of any effective learning program, and standardization of these components can help ensure that training is effective, efficient, and consistent and will allow evaluation of its impact. Impact is best identified through a range of evidence that provides robust verification for enhanced knowledge, behaviors, and practices, Furthermore, researchers are advised to consider both quantitative and qualitative forms of evidence. A longitudinal approach to evaluation is recommended in preference to singular measurements to ensure that the full value of an intervention can be captured over time and skills retention is measured. Program designers may also wish to consider evaluation at both participant and institutional level.

### Faculty Development

Verge et al [42] stated that it is crucial to enhance the teaching staff’s required qualifications, account for institutional opposition to gender-related change, and implement monitoring and evaluation systems. These steps are important to ensure that training outcomes are regularly assessed and improved when integrating gender equality training into higher education programs.

### Compulsory Training

The United Nations Educational, Scientific and Cultural Organization [1] recommends that gender equality should be integrated into the whole program of faculties of education, rather than being included as a course. Toraman and Özen [14] found that the gender equality training offered as a compulsory-elective course in the faculty of education did not produce the expected results for their students. De Villers et al [39] noted that student leaders who volunteer may have leadership value systems and may not reflect the student body. Abrams et al [31] indicated plans to embed their program in the engineering curriculum and participants would be recruited as future teaching assistants to enhance sustainability. It has been demonstrated that elective courses in general receive more favorable responses than the required courses using both scaled response evaluation formats and open‐ended response evaluation forms [43] and these observations have relevance for gender equality training. When participation is voluntary, those who opt in are likely to have a preexisting interest, motivation, or alignment with the course’s objectives, which can positively influence their engagement and satisfaction. Self-selection bias may skew evaluations and limit the generalizability of findings regarding the course’s effectiveness. In addition, voluntary participants are typically intrinsically motivated, which enhances their willingness to engage deeply with course material and reflect on their learning. In contrast, students who are required to take part in such courses may approach the material with skepticism, resistance, or apathy, potentially lowering their engagement and perceptions of the course [44].

Mandating participation can broaden the reach of training; however, course evaluations should account for differences in participant motivations. Care should be taken to ensure that the course’s status as elective or required is considered for evaluation purposes [43]. Combining qualitative and quantitative assessment methods can provide a more nuanced understanding of the course’s impact, particularly on participants who might initially be less engaged. Furthermore, longitudinal studies tracking behavior change or attitude shifts after training can help determine the broader efficacy of these programs beyond immediate satisfaction ratings [45].

Lau et al [46] proposed that a key reason for the lack of progress in gender parity in organizations lies in the predominance of empirical research focusing on the causes and manifestations of gender inequality, while insufficient attention has been given to exploring solutions. To advance gender equality, they argue that a paradigm shift from problems to solutions is critical and urgent.

Significant research is required to bring gender mainstreaming to higher education. The Swedish Secretariat for Gender Research [47] distinguishes between gender-mainstreamed teaching as a pedagogical practice and the integration of gender and gender equality knowledge into the subject content. They call for resources for research-based pedagogical development for the implementation of teaching activities.

### ToC Framework

#### Overview

Applying the ToC framework can enhance the practical value of our findings, offering a structured approach for educators to better understand the pathways through which gender equality training can lead to desired outcomes. Viewed through the ToC lens, the findings from this review demonstrate the importance of aligning gender equality training programs with a clear, structured pathway from inputs and activities to outcomes and impact. To achieve transformative change in attitudes, behaviors, and institutional practices, the following elements emerge as critical:

#### Inputs and Activities

Effective programs must begin with carefully designed interventions rooted in robust pedagogical frameworks. These interventions should actively engage participants, address intersectional perspectives, and incorporate diverse methodologies, such as service-learning or active, immersive simulation-based activities, and leadership-focused training.

#### Outputs

Outputs should be aimed at improving participants’ awareness, knowledge, and attitudes toward gender equity. Training activities must also equip participants with practical skills, such as leadership and communication skills, and encourage and motivate the application of these skills.

#### Outcomes

The outcomes from training programs should focus on fostering shifts in individual behaviors and attitudes, including a heightened ability to identify and address implicit biases, privileges, and microaggressions. Critical success outcomes would include embedding these skills and behaviors within institutional practices to promote systemic change.

#### Impact

To move toward the desired systemic impact of gender equality within higher education and beyond, interventions need to demonstrate sustainable behavior changes at the organizational and societal levels. Embedding gender equity principles into institutional structures—such as curriculum design, faculty development, and organizational policies—will help to create a ripple effect, influencing broader cultural norms and reducing structural barriers to equality.

#### Assumptions and Contextual Factors

The success of training interventions depends on several assumptions, such as participant willingness to engage, institutional buy-in, and adequate resources for program implementation and evaluation. The evidence suggests that voluntary participation may enhance learner engagement, but care must be taken to mitigate self-selection biases by complementing elective offerings with strategically designed mandatory components.

To bridge the gap between current practice and desired outcomes, a ToC-driven approach should prioritize longitudinal, multilevel evaluations that measure not only immediate knowledge gains but also the sustainability and transferability of learning outcomes. This includes tracking participants’ ability to influence institutional and societal change over time. By connecting theory, evidence, and practice, this framework provides a cohesive road map for designing and implementing impactful gender equality training programs in higher education.

### Strengths and Limitations

The search strategy was comprehensively developed by all authors with the support of an experienced librarian to facilitate a thorough and extensive database search. The searches carried out were limited to the 9 databases available to the authors. However, following consultation with the librarian, the process of hand-searching the reference lists of the included articles to identify important studies and gray literature may be considered a strength of this review. It is also important to note the lack of relevant literature available, highlighting a significant gap in the evidence base for this area and this should encourage further research. Considering the accelerating interest in equality, diversity, and inclusion issues in most recent years, relevant work may have been missed in the months since the main literature search was conducted. Excluding studies which focused on mentorship programs, gender equality in health care delivery, and gender studies modules may have excluded potentially useful information. However, following the preliminary database searching and screening, we concluded that these studies did not fully align with the research objectives for this review.

### Implication for Practice

This review aimed to map the depth and breadth of gender equality training for student leaders in HEI to support curriculum development for training. Our findings suggest room for improvement in the conduct and reporting of research on training interventions with particular attention to theoretically informed decisions about the development of learning activities, the choice of instructional methods, and tools and resources to implement the interventions. In addition, a more effective approach to evaluation that goes beyond the immediate reaction of learners and assesses behavior change is required to allow continuous improvements in this field. Educational programs for gender equality can play a significant role in fostering awareness, knowledge, and skills necessary to address gender disparities in the higher education sector. However, they cannot operate in isolation. There is a real risk that stand-alone educational initiatives will fail to create lasting, systemic change if they are not integrated into a broader, multipronged strategy that addresses the structural, cultural, and institutional barriers perpetuating gender inequality [40].

### Conclusions

Initiatives such as the Athena Swan Charter, a framework to support gender equality within higher education and research, seek to advance equal opportunities for all genders in HEIs across the globe. However, significant gender inequality remains and encouraging positive leadership among students may help to build capacity to support equality. Appropriate sustainable and effective skills training is needed to increase awareness and nurture competencies for male, female, and nonbinary student leaders to actively address gender inequality. Investments in gender equity training demonstrate organizational commitment to inclusivity. Long-term dedicated financial support is essential for the sustainability of training interventions aimed at promoting gender equity. Adequate funding enables the development of high-quality training materials, the engagement of skilled facilitators, and the integration of innovative methodologies. Funding also facilitates the evaluation and monitoring of programs, ensuring that interventions remain effective and adaptable to changing needs and contexts. Without sustainable funding, training programs risk becoming fragmented or short-lived, limiting their impact on fostering institutional change.

## Appendix

Multimedia Appendix 1 Preferred Reporting Items for Systematic reviews and Meta-Analyses extension for Scoping Reviews (PRISMA-ScR) Checklist.

## Abbreviations

EIGE: European Institute for Gender Equality
HEI: higher education institution
ToC: theory of change
